# A case of a young man with cutaneous horn arising from underlying dermatofibroma and its dermoscopic features

**DOI:** 10.1016/j.jdcr.2025.02.030

**Published:** 2025-03-12

**Authors:** Nello Tommasino, Luigi Guerriero, Lorenzo Scaramuzzino, Massimiliano Scalvenzi, Antonio Portarapillo

**Affiliations:** Section of Dermatology, Department of Clinical Medicine and Surgery, University of Naples Federico II, Naples, Italy

**Keywords:** dermatofibroma, dermoscopy, horn, tumor

## Clincial presentation

**Fig 1:**
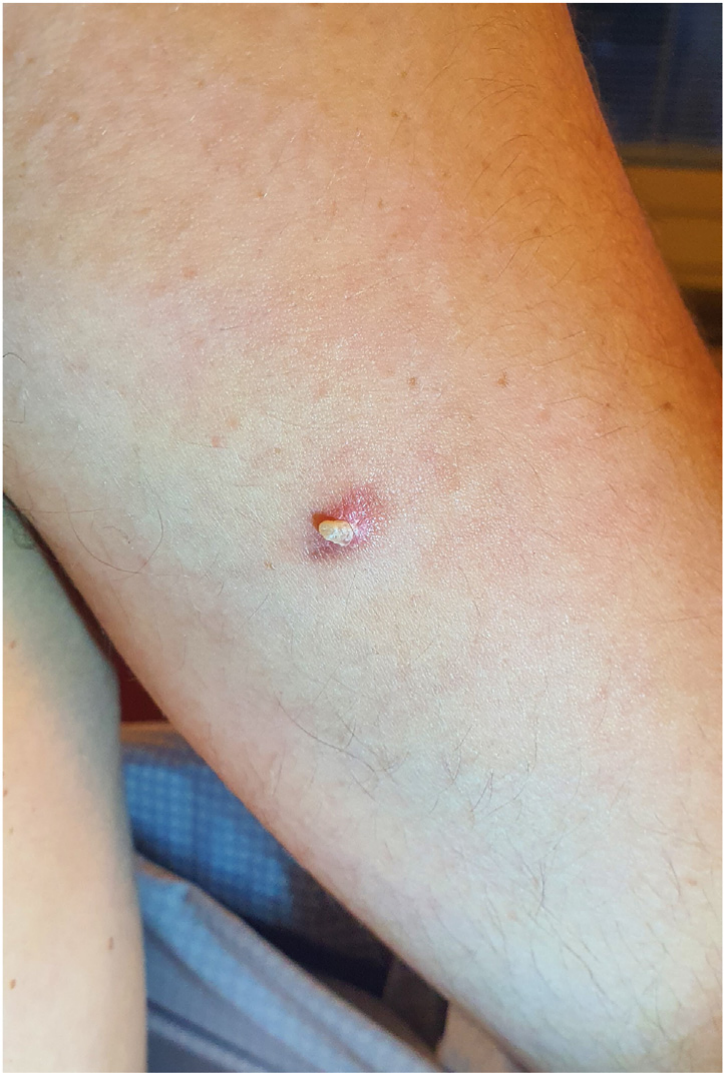
Pinkish-brownish left arm papule surmounted centrally by a hyperkeratotic cone.

A 29-year-old man presented on his left arm with a brownish-pink, 0.5 cm-diameter, hard, asymptomatic papule surmounted by a hyperkeratotic cone that had been growing in the central area for about 1 year (Fig 1).

## Dermoscopic appearance

**Fig 2:**
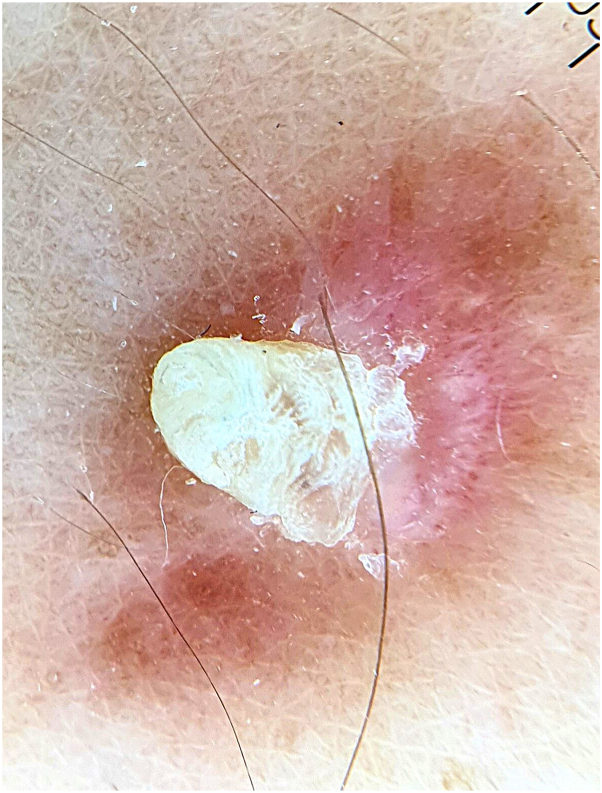
Dermoscopic features including whitish-pink area with dotted vessels surrounded by a brownish peripheral network and surmounted by a yellowish cone.

Dermoscopy revealed the presence of a central yellowish conical projection, surrounded by a whitish-pink area with dotted vessels, which in turn was surrounded by a brownish peripheral network (Fig 2).

## Histological diagnosis

Histologic examination showed hyperplastic and hyperkeratotic epidermis in the central area, with circumscribed dermal proliferation of CD10^+^ fusiform cells, compatible with the diagnosis of dermatofibroma surmounted by cutaneous horn in the central region.

**Keypoints**

Dermatofibroma is a benign skin tumor characterized by proliferation of histiocytes or spindle cells in the dermis.^1^ Generally, it requires no treatment. Sometimes doubtful forms should be distinguished from other skin cancers such as melanoma, basal cell carcinoma, and squamous cell carcinoma.^2^ Dermatofibroma may also rarely be associated with an overlying cutaneous horn, as in the only case reported in literature described by Nam et al.^1^ It is a hyperkeratotic protrusion, the pathogenesis of which is not fully elucidated, typical of the elderly age and which may be associated with malignant transformation into squamous cell carcinoma.^1^ In the case described, the lesion was excised for this risk and because the horn masked the dermoscopic features of the lesion, which could also appear as a Bowen disease. The aim of the report is to describe a rare case of a young patient with cutaneous horn arising on dermatofibroma and emphasize when its excision is indicated.

## Conflicts of interest

None disclosed.
